# Peroxisome Proliferator-Activated Receptors Associated with Nonalcoholic Fatty Liver Disease

**DOI:** 10.1155/2017/6561701

**Published:** 2017-12-05

**Authors:** Nan Wang, Rui Kong, Hui Luo, Xiaorong Xu, Jie Lu

**Affiliations:** ^1^Department of Gastroenterology, Shanghai Tenth People's Hospital, Tongji University School of Medicine, Shanghai 200072, China; ^2^The School of Medicine of Soochow University, Suzhou 215006, China

## Abstract

Nonalcoholic fatty liver disease (NAFLD) is rapidly becoming a major cause of chronic liver disease worldwide. Concurrent to an increase in NAFLD prevalence, there is an increase in the obesity epidemic and the correlated insulin-resistant state. It is a challenge to diagnose NAFLD because many patients are asymptomatic until the later stages of disease. The most common symptoms include fatigue, malaise, and discomfort in the right upper quadrant. The major and most accurate tool to clinically diagnose NAFLD is a liver biopsy, followed by histological analysis. However, this procedure is invasive and often carries a high risk of complications. Currently, there are no officially approved medications for the treatment of NAFLD. Although lifestyle modifications with proper diet and exercise have been shown to be beneficial, this has been difficult to achieve and sustain for many patients. Effective pharmacological treatments are still lacking; therefore, additional research to identify novel drugs is clearly warranted. PPARs are promising drug targets for the management of NAFLD and its related conditions of type 2 diabetes mellitus and cardiovascular disease. In this review, we provide an overview of recent studies on the association of PPARs and NAFLD.

## 1. Introduction

Nonalcoholic fatty liver disease (NAFLD) has been defined as hepatic steatosis without significant alcohol intake or other diseases [1]. It also has been defined as the hepatic manifestation of metabolic syndrome (MS), whose main symptoms include insulin resistance state, atherogenic dyslipidemia, abdominal obesity, and hypertension [1]. Currently, there is growing evidence that NAFLD is a multisystem disease, affecting extrahepatic organs [2]. NAFLD increases the risk of type 2 diabetes mellitus (T2DM), chronic kidney disease (CKD), and cardiac diseases, including cardiovascular disease (CVD) [2]. The pathological state of NAFLD results from high levels of circulating free fatty acids (FFAs), leading to accumulation of lipid deposits within hepatocytes that triggers steatosis. The clinical spectrum of NAFLD ranges from simple steatosis to steatohepatitis, bridging fibrosis, and cirrhosis [3]. The primary step in NAFLD is triglyceride accumulation in hepatocytes, which appears to be dependent on IR [4]. The second state involves hepatocellular injury, namely, nonalcoholic steatohepatitis (NASH), which includes many factors, including oxidative stress, increased proinflammatory cytokines, mitochondrial dysfunction, iron overload, bacterial overgrowth, and genetic predisposition [5]. Many NAFLD patients ultimately progress to liver cirrhosis and hepatocellular carcinoma [6, 7]. In recent studies, the pathophysiology of NAFLD was reported, including the theories of “two-hit” and “multiple parallel hit” [8, 9]. However, the precise mechanism involved in the development and progression of NAFLD is not completely understood.

## 2. Peroxisome Proliferator-Activated Receptors

### 2.1. PPARs Overview

Peroxisome proliferator-activated receptors (PPARs) are an important group of receptors that play a role in mediating the pleiotropic effects of diverse environmental contaminants, food components, and drugs [10]. PPARs are members of the nuclear receptor superfamily and induce the expression of numerous genes by functioning as ligand-activated transcription factors. The ligands of several PPARs include serial compounds and endogenous lipids, such as FFAs and eicosanoids [11]. Once a ligand binds to the promoter of the target gene, a heterodimer is formed with the retinoid X receptor (RXR). This heterodimer subsequently binds response elements that regulate the expression of genes encoding enzymes or proteins involved in beta oxidation, fatty acid (FA) uptake, adipogenesis, adipocyte differentiation, ketogenesis, bile acid synthesis, triglyceride turnover, and IR [12, 13].

In mammals, three PPAR isoforms have been identified: alpha (*α*), beta/delta (*β*/*δ*), and gamma (*γ*), which are differentially distributed in various tissues [11]. PPAR*α* is ubiquitously expressed and is primary found in the liver, heart, and kidneys. In addition, PPAR*β*/*δ* is also ubiquitously expressed in muscle, adipose tissue, and the liver. PPAR*γ* has three isoforms (*γ*1, *γ*2, and *γ*3) that display differences in tissue localization for each isoform; for example, *γ*1 has a ubiquitous tissue localization, *γ*2 is primarily localized in adipose tissue, and *γ*3 is localized in macrophages, colon, and adipose tissue [11, 14–17]. PPARs function as transcription factors that regulate the expression of genes involved in adipose metabolism, glucose metabolism, and cellular proliferation and differentiation (Table 1) [11, 15–17].

**Figure 1:**
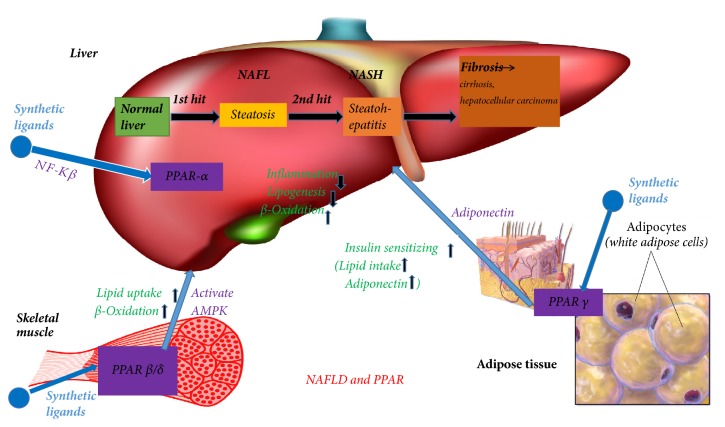
The relation between PPAR and NAFLD.

Previous studies have identified various roles for PPAR*δ* in inflammation, lipid metabolism, and cancer [18]. Because of its intrinsic expression and diverse cellular actions, no single descriptor appropriately describes the biological function of PPAR*δ*P. PPAR*γ* is known as the critical transcriptional regulator of the induction of adipogenesis [19]. This process occurs in fat cells that differentiate from preadipocytes into mature adipose cells. In addition to adipocytes, PPAR*γ* is also expressed with a limited number of other cell types to cause anti-inflammatory actions and promotes lipid storage [20]. PPAR*γ* is the molecular target of the insulin-sensitizing drugs pioglitazone and rosiglitazone, so PPAR*γ* has been recognized as one of the key receptors in the pharmacological treatment of T2DM [21]. PPAR*α* has an important role in the liver and acts as the main regulator of lipid metabolism, particularly during fasting [22–24]. Fasting is associated with lipid homeostasis in the liver, which is coordinated by PPAR*α*. Previous studies using low and high throughput gene expression analysis have demonstrated that PPAR*α* modulates the expression of numerous genes involved in virtually every profile of lipid metabolism, including FFA uptake, mitochondrial and peroxisome fatty acid oxidation (FAO), cytogenesis, and the formation and breakdown of triglycerides and lipid droplets [25]. Therefore, in this review, we aimed to summarize recent advancements that have been reported on PPAR-related genes and target drugs, thereby promoting the molecular mechanism of action of NAFLD (Figure 1).

### 2.2. PPAR Gene Polymorphisms

Three distinct forms of the PPAR family exist in humans and rodents and all are encoded by different genes that have been identified and well characterized. The gene for PPAR*α* is* NR1C1*, the gene for PPAR(*β*/*δ*) is* NP1C2*, and the gene for PPAR*γ* is* NR1C3* [14]. All three PPARs are heterodimers that bind to DNA using retinoid X receptors (RXR:NR2B subgroup). They preferentially bind to direct repeats of the nuclear receptor half site AGGTCA separated by 1 nucleotide (DR1).

Each subtype has unique functions. PPAR binds to direct repeats of the nuclear receptor half site AGGTCA separated by 1 nucleotide (DR1) independently. The PPARs gene is located on chromosome 3 and is linked to NAFLD. The C/G polymorphism (rs1801282) results in a Pro-to-Ala change, which represents a substitution of proline (Pro) with alanine (Ala) at codon 12. It was found that this substitution is related to a decrease in both DNA binding and transcriptional activity, and as a result, the encoded Ala allele form is reduced. The Pro12Ala variant is associated with increased insulin sensitivity, a lower body mass, and protection from T2DM. Carriers of the PPAR*γ* Ala allele demonstrate increased resistance to NAFLD development and progression by inhibiting oxidative stress [26, 27]. Additionally, a meta-analysis study revealed a protective role against NAFLD for the Ala allele of the PPAR*γ* Pro12 Ala (rs1801282) polymorphism [28]. Moreover, the rs1801282 polymorphism was associated with NAFLD susceptibility in East Asians but not in European populations [28]. Domenici et al. (2013) showed that the 12 Ala allele of PPAR*γ* was less prevalent among NASH patients compared to the healthy volunteers group. No associations were found among PPAR*γ* single nucleotide polymorphisms (SNPs) (rs1801282) and clinical, laboratory, and histological parameters in NAFLD patients. However, it was shown that the rs1801282 SNP may lead to protection against liver injuries [29]. The PPAR*α* SNP Leu162Val (rs1800206) may be involved in the progression of NAFLD because carriers of this SNP have more advanced fibrosis [29]. However, Wang et al. suggested that the rs1801282 polymorphism of PPAR*γ* was not associated with NAFLD risk in both Asian and Caucasian descents based on a meta-analysis study [30]. However, the rs1800206 polymorphism of PPAR*α* showed to be associated with lipoprotein (a) (Lp(a)). Lp(a) is a low density lipoprotein- (LDL-) like particle that is associated with increased risk of atherosclerosis and CVD [31, 32]. Thus, it provides evidence that PPAR*α*/*γ* may influence the risk of dyslipidemia and CVD via Lp (a) [31, 32]. The Ala12Ala genotype of PPAR*γ*2 may decrease the number of diseased vessels and the severity of CVD. This may be due to a direct antiatherogenic effect of this polymorphism, as well as an indirect effect through its association with reduced inflammatory parameters and IR [33]. The SNP rs3856806 (also termed C161T or C1431T) in PPAR*γ* was significantly associated with a fasted serum lipid profile [34]. Wan et al. suggested that the PPAR*γ* SNP rs3856806 may reduce the risk of severe atherogenesis by modulation of the adipose metabolism in Chinese patients with CVD [35]. Previous studies have shown that the SNP rs3856806 increased NAFLD susceptibility through the adiponectin pathway [36, 37].

Moreover, recent studies indicated that fibroblast growth factor 21 (FGF21) functions as an endocrine hormone that mediates many of the effects of PPAR*α*. PPAR*α* directly induced the gene encoding FGF21 during fasting via a binding site in the promoter. Subsequently, FGF21 stimulated lipolysis in adipose tissue and cytogenesis in the liver.* In vitro* studies demonstrated that fatty acids act as ligands that can bind PPAR*α*.

G0/G1 switch gene 2 (G0S2) plays a significant role in controlling lipolysis in adipocytes and serves as a target gene of PPARs. It was first found to be differentially expressed in lymphocytes during the lectin-induced switch from the G0 to G1 phase of the cell cycle [38, 39]. The highest levels of G0S2 mRNA are found in brown and white adipose tissue and are largely upregulated during adipogenesis in mouse 3T3-L1 cells [40]. These data indicate that G0S2 may participate in lipid metabolism. Recently, Yang et al. reported that G0S2 localizes to lipid droplets and prevented adipose triglyceride lipase- (ATGL-) mediated turnover occurrence in HeLa cells and adipocytes [41]. Both human and mouse G0S2 genes encode a protein of 103 amino acids and have 78% sequence homology [42]. Additional studies have revealed that G0S2 mRNA is highly expressed in brown and white adipose tissue and is upregulated during growth arrest in 3T3-L1 fibroblasts [40]. The activation of PPAR*γ* and PPAR*α* [40], PPAR*β*/*δ* [43], retinoic acid [42, 44], and IR can upregulate G0S2 expression in 3T3-L1 cells and human acute promyelocytic leukemia cells [41]. Furthermore, Ong et al. reported that G0S2 inhibits the triglyceride hydrolase activity of ATGL, which is a major regulator of the lipid metabolism in mammals [45]. ATGL is also a major lipase in the liver, which indicated that G0S2 may have a significant role in regulating lipolysis in adipocytes [41].

## 3. PPAR*α*

PPAR*α* is important in the regulation of lipid transport and metabolism chiefly through the activation of mitochondrial and peroxisomal fatty acid *β*-oxidation pathways. PPAR*α* regulates the transcription of constitutive genes encoding fatty acid-metabolizing enzymes and mitochondrial FA oxidation (FAO) activity, primarily in the liver [46]. PPAR*α* directly inhibits inflammatory genes induced by NF-*κ*B [47] and reduces IL-1-induced expression of C-reactive protein in human primary hepatocytes [48]. Moreover, fenofibrate treatment decreased the IL-6-induced expression of acute phase response genes in the livers of wild type mice but not PPAR*α*-deficient mice. Fenofibrate treatment also reduced plasma concentrations of the acute phase proteins fibrinogen, C-reactive protein, serum amyloid A, plasminogen, and alpha 2-macroglobulin in human subjects [49–51]. PPAR*α* activators, such as the widely prescribed fibrate drugs, ameliorate hepatic steatosis by enhancing mitochondrial FAO in mice [52]. Furthermore, PPAR*α* has an anti-inflammatory role after being fed with a high-fat diet (HFD) [53]. PPAR*α* is a transcriptional regulator of the lipid pathway and a direct target of a microRNA (miRNA or miR) in the liver (miR-34a). The miR-34a-PPAR*α* has recently been identified and provides a novel clue to the pathogenesis of steatosis in NAFLD [54]. In fact, miR-34a may become a target for the treatment of NAFLD.

Currently, it is suggested that microRNAs contribute to the pathogenesis of NAFLD. MicroRNAs act as potent posttranscriptional regulators of gene expression [55, 56]. MiR-21 is increased in the liver of NASH patients [49] and in animal models of NASH [54, 57–59]. In hepatocytes, miR-21 is induced by unsaturated fatty acids in an mTOR/NF-*κ*B-dependent manner [60]. In addition, recent studies have demonstrated that hepatic miR-21 plays an active role in NASH pathogenesis by inhibiting PPAR*α*, which is a key target of miR-21 [54]. The miR-21/PPAR*α* axis regulates PPAR*α* in muscle tissue of NAFLD patients, which may be important in disease development and progression.

Similarly, PPAR*α* is activated by a wide range of different fatty acids and eicosanoids [61–65]. Moreover, PPAR*α* served as a receptor for various synthetic compounds, collectively referred to as peroxisome proliferators [12]. This includes phthalates, insecticides, herbicides, surfactants, organic solvents, and hypolipidemic fibrate drugs. For decades, fibrates have been used by divers primarily because of their ability to decrease circulating triglycerides [66]. The first generation of PPAR*α* agonists Clofibrate and Fenofibrate have been thought to primarily activate PPAR*α* in the liver, resulting in improvements in the plasma lipid profile. However, it is concerning that the World Health Organization conducted a clinical trial in which increased coronary and overall mortality rates in the Clofibrate treatment groups were detected [67]. Bezafibrate is a low potency compound that activates all three PPAR isoforms. It has been shown to decrease the risk of cardiovascular events and prevent the onset of diabetes in patients with metabolic syndrome [68]. It has also been reported to be safe and well tolerated [69]. Thus, pharmacological targeting of PPAR*α* has been considered as a promising treatment of NAFLD.

A dual PPAR*α*/*δ* agonist called GFT505 has been indicated to promote markers of liver dysfunction, reduce hepatic lipid accumulation, and decrease hepatic inflammatory gene expression in liver in numerous animal models of NAFLD [60]. Moreover, GFT505 treatment reduced liver dysfunction markers and enhanced hepatic and peripheral insulin sensitivity in humans [60, 61].

## 4. PPAR*δ*

Pharmacological targets of PPAR*α* and PPAR*γ* are relatively well-known in the therapies of dyslipidemia and diabetes [70]. However, PPAR*δ* also has a significant role in the regulation of metabolism since its activation augments fatty acid oxidation, alleviates glucose homeostasis, and reduces macrophage inflammatory responses [15, 70]. According to a current study, the PPAR*δ* agonist GW0742 was suggested to reduce the renal dysfunction and inflammation caused by chronic high-fructose corn syrup (HFCS-55) exposure by preventing the activation of the NLRP3 inflammasome in the nephridium [71]. In a recent study, it was suggested that GW501516 decreased activation of the inflammasome and overproduction of proinflammatory cytokines in HepG2 cells and mouse livers [72]. Furthermore, GW501516 alleviated hepatic steatosis* in vivo*.

Due to its ability to maintain metabolic homeostasis, AMPK is an important metabolic regulator in cellular and organismal survival and an essential mediator for fatty acid metabolism [72–74]. Numerous studies have demonstrated that PPAR*δ* prevented the downregulation of AMPK [72, 73]. PPAR*δ* agonist GW501516 inhibited activation of the inflammasome and reduced IL-1*β* levels. Additionally, GW501516 potentially modulated AMPK phosphorylation and reduced the production of reactive oxygen species. This anti-inflammatory effect may be related to the amelioration of hepatic steatosis in mice. The target of the inflammasome by the PPAR*δ* agonist might be related to the therapeutic implications for the treatment of NAFLD.

Lastly, a novel PPAR*δ* agonist, MBX-8025, was evaluated in a small, randomized, double-blind, placebo-controlled study. This study included overweight subjects with dyslipidemia and found that treatment with MBX-8025 resulted in favorable lipid profiles and decreased gamma-glutamyl transpeptidase (GGT) [75]. However, other markers of NAFLD or liver injury were not measured in this study, which has limited the interpretation of the treatment effectiveness of NAFLD.

## 5. PPAR*γ*

The PPAR*γ* coactivator 1 (PGC1) is a group of transcriptional coactivators that include PGC1*α*, PGC1*β*, and the PGC related coactivator (PRC) [76]. PGC1*α* is related to transcriptional factors such as PPAR*α*, PPAR*γ*, estrogen-related receptor, liver X receptor, and hepatocyte nuclear factor-4*α*. Furthermore, PGC1*α* regulates the mitochondrial metabolism [77] and modulates energy, glucose, and adipose metabolism. These characteristics are recognized as key therapeutic targets for diabetes and obesity. A recent study in humans also suggested that the mRNA expression of PPAR*γ* was markedly higher in obese patients (*n* = 22, NAFLD) when compared to controls. Moreover, PPAR*γ* expression in the liver was suggested to have a positive correlation with sterol regulatory element binding protein 1c mRNA levels, serum insulin levels, and homeostasis model assessment-insulin resistance and has a negative correlation with total adiponectin levels [78]. Thus, it was determined that the peroxisome proliferator-like effects of rosiglitazone in fat mice might be due to the activation of PPAR*γ*. In a recent study, it was suggested that palmitate (not oleate) upregulates PPAR*γ* via PGC1s in Huh7 cells. Moreover, both PGC1*α* and PPAR*γ* are similarly upregulated and palmitate constituent was increased in the liver in the NAFLD mouse model, indicating a positive correlation with the triglyceride content. This suggested an explicit effect on the lipid metabolism that causes intrahepatic triglyceride accumulation.

It is well established that PPAR*γ* is the primary regulator of adipose tissue development and function and is more abundant in adipose tissue than in any other cell type. Thiazolidinediones (TZDs) play a key role in stimulating progenitor stem cells to differentiate in adipocytes and influence mature adipocytes [79]. Current reports have also shown that TZDs enhance FGF21 expression in adipose tissue. TZDs also increase PPAR*γ* transcriptional activity in an autocrine or paracrine manner and target PPAR*γ* by posttranslational modification. TZDs represent a class of clinically used insulin-sensitizing drugs, which currently includes rosiglitazone and pioglitazone that exert many other pleiotropic effects [79]. Pioglitazone has shown to improve endothelial dysfunction, reduces blood pressure, corrects diabetic dyslipidemia, and reduced circulating levels of inflammatory cytokines and prothrombotic factors [79]. Consistent with these antiatherogenic effects, pioglitazone reduced major adverse cardiac endpoints such as mortality, myocardial infarction, and stroke [80].

Most studies on PPAR*γ* ligands have focused on agonists. Here, we identified that isorhamnetin, a natural and novel identified compound in fruits, vegetables, and the metabolite of quercetin, ameliorated MS induced by diet or leptin deficiency [81]. Isorhamnetin treatment inhibited 3T3-L1 adipocyte differentiation induced by PPAR*γ* agonists such as rosiglitazone [81, 82]. It can reduce the expression of genes downstream of PPARs and antagonize PPAR*γ* transactivity. Moreover, it also reduced the onset of obesity and ameliorated hepatic steatosis induced by both high-fat diet treatment and leptin deficiency [83–85]. In addition, it was also indicated that gypenoside treatment significantly reduced levels of PPAR*γ*, thereby having potential hepatoprotective effects [86]. These results suggested that dietary supplements of isorhamnetin may be beneficial for the prevention of obesity and steatosis. In addition, PPARi antagonists may be a promising therapy for hepatic steatosis.

## 6. Conclusions

In this review, several molecular pathways have been outlined, contributing to therapies for NAFLD. However, the precise details of these pathways are still being elucidated, but they provide targets for future therapeutic strategies in NAFLD treatment. In addition, the efficacy and safety of NAFLD management should be considered. Additional clinical evidence is required before their clinical application, and further large-scale clinical trials and practice are warranted to verify drug treatment effects.

## Figures and Tables

**Table 1:** PPARs expression and respective ligands.

Types	Major distribution	Targets
*Agonist*		

PPAR*α*	Liver, heart, kidney	Fenofibrate
		Clofibrate
		(FGF21)
		(G0S2)

PPAR*β*/*δ*	Skeletal muscle, liver	GW0742
		GW501516
		MBX-8025

PPAR*γ*	Adipose tissue	TZDs
		(rosiglitazone, pioglitazone)
		Palmitate
		PGC1

PPAR*α*/*δ*		GFT505 (elafibranor)

PPAR*α*/*γ*		Tesaglitazar
		Glitazars (saroglitazar)

*Antagonist*		

PPAR*γ*		Isorhamnetin (quercetin)

PPAR*α*/*γ*		Z-551
